# Parental cigarette smoking and childhood risks of hepatoblastoma: OSCC data

**DOI:** 10.1038/sj.bjc.6601651

**Published:** 2004-03-02

**Authors:** T Sorahan, R J Lancashire

**Affiliations:** 1 Institute of Occupational Health, University of Birmingham, Edgbaston, Birmingham B15 2TT, UK; 2 Department of Public Health and Epidemiology, University of Birmingham, Edgbaston, Birmingham B15 2TT, UK

**Keywords:** hepatoblastoma, childhood cancer, parental smoking

## Abstract

Reported cigarette smoking habits for the parents of 43 UK children who died with hepatoblastoma (1953–55 deaths, 1971–81 deaths) have been compared with corresponding information for the parents of 5777 healthy control children by means of unconditional logistic regression. Hepatoblastoma risks were doubled if both parents smoked relative to neither parent smoking (RR 2.28, 95% CI 1.02–5.09).

Three reports from the Oxford Survey of Childhood Cancers have suggested that paternal but not maternal cigarette smoking is associated with increased risks for the generality of childhood cancers (Sorahan *et al*, 1995, 1997a, 1997b). Other studies, however, have produced conflicting findings (Thornton and Lee, 1998; Sorahan *et al*, 2001) and, for example, the large study reported recently from the United Kingdom Childhood Cancer Study (UKCCS) found no important positive effects for paternal smoking and the risks of all childhood cancers (Pang *et al*, 2003). A significant association was reported in this latter study, however, between hepatoblastoma risks and smoking by both parents relative to neither parent smoking (RR 4.74 95% CI 1.68–13.35, *P*=0.003). OSCC data have been examined, therefore, to provide additional information on any relation between parental cigarette smoking and hepatoblastoma.

## MATERIALS AND METHODS

The OSCC, a national case–control study into the aetiology of childhood cancer, began in Oxford in 1955, but has been located at the University of Birmingham since 1975. The survey sought to interview the parents (usually the mother) of all children dying of cancer before their 16th birthday in England, Wales and Scotland for the period 1953–84. A number of standard questionnaires, covering a wide range of social and medical topics, were used during the course of this prolonged study. Data on parental cigarette smoking were not collected for all years of the study, but sought for 1953–55 deaths, 1971–76 deaths and 1977–81 deaths (and matched controls). The survey and the information available on smoking histories have been described previously (Stewart *et al*, 1958; Gilman *et al*, 1988; Sorahan *et al* 1995, 1997a, 1997b).

A total of 5777 matched case–control pairs (all diagnoses) with smoking histories were available for analysis (Sorahan *et al*, 1997b). The abstracts of hospital records collected contemporaneously have been reviewed for all 64 liver tumours among which 43 hepatoblastomas were identified. Case and control data relating to smoking histories were then compared by means of unconditional logistic regression using the EGRET program. The use of unconditional logistic regression enabled comparisons to be made between the cases of hepatoblastoma (*n*=43) and the entire series of controls for whom smoking details were available (*n*=5777). Relative risks (odds ratios) for categories of parental smoking were first estimated without adjustment for other variables. These analyses were then repeated with adjustment for three of the four original matching variables (sex of child, age at death or corresponding age for controls, year of death or corresponding year for controls). Computerised information on region of residence was not available for these analyses. Further analyses also adjusted for social class, sibship position, maternal age at birth of child, paternal age at birth of child and obstetric radiography. The procedures adopted to code social class have been described previously (Lancashire and Sorahan, 2003). The smoking histories are those analysed previously (Sorahan *et al*, 1997b) except that ex-smokers who gave up at least 2 years before the birth of the survey child have now been combined with the nonsmokers; ex-smokers who gave up either shortly before the birth or after the birth are classified as smokers.

## RESULTS

Number of cases (hepatoblastoma and all diagnoses) and controls are shown by categories of parental smoking habits in Table 1

**Table 1:** Relative risks of hepatoblastoma and all childhood cancers in relation to parental cigarette smoking: 5777 OSCC matched pairs (1953–55 deaths, 1971–76 deaths, 1977–81 deaths)

					**RR^a^**	**(95% CI)**	**RR^b^**	**(95% CI)**	**RR^c^**	**(95% CI)**
**Diagnosis**	**Parental cigarette smoking**		**Cases (n)**	**Controls (n)**	**unadjusted**		**adjusted for matching variables**		**additional adjustments**	
*A. Separate analyses of maternal and paternal smoking habits*										
All cancers	maternal	non-smoker	3091	3191	1.0		1.0		1.0	
		smoker	2616	2524	1.07	(0.99 to 1.15)	1.07	(0.99 to 1.15)	1.06	(0.98 to 1.14)
		not known	70	62	—		—		—	

	paternal	non-smoker	1975	2267	1.0		1.0		1.0	
		smoker	3601	3359	1.23***	(1.14 to 1.33)	1.25***	(1.15 to 1.35)	1.27***	(1.17 to 1.38)
		not known	201	151	—		—		—	

Hepatoblastoma	maternal	non-smoker	19	3191	1.0		1.0		1.0	
		smoker	24	2524	1.60	(0.87 to 2.92)	1.62	(0.88 to 2.98)	1.73	(0.93 to 3.21)
		not known	0	62	—		—		—	

	paternal	non-smoker	12	2267	1.0		1.0		1.0	
		smoker	28	3359	1.58	(0.80 to 3.10)	1.87	(0.93 to 3.74)	2.10*	(1.03 to 4.25)
		not known	3	151	—		—		—	

*B. Simultaneous analyses of maternal and paternal cigarette smoking habits*										
All cancers	maternal	non-smoker	3091	3191	1.0		1.0		1.0	
		smoker	2616	2524	1.02	(0.94 to 1.10)	1.02	(0.94 to 1.10)	1.01	(0.93 to 1.09)
		not known	70	62	—		—		—	

	paternal	non-smoker	1975	2267	1.0		1.0		1.0	
		smoker	3601	3359	1.23***	(1.13 to 1.33)	1.24***	(1.14 to 1.35)	1.27***	(1.17 to 1.38)
		not known	201	151	—		—		—	

Hepatoblastoma	maternal	non-smoker	19	3191	1.0		1.0		1.0	
		smoker	24	2524	1.44	(0.77 to 2.68)	1.40	(0.75 to 2.62)	1.47	(0.78 to 2.77)
		not known	0	62	—		—		—	

	paternal	non-smoker	12	2267	1.0		1.0		1.0	
		smoker	28	3359	1.44	(0.72 to 2.89)	1.68	(0.82 to 3.45)	1.88	(0.91 to 3.89)
		not known	3	151	—		—		—	

*C. Simultaneous analyses of maternal and paternal cigarette smoking habits (alternative approach)*										
All cancers	neither parent		1385	1601	1.0					
	mother only		585	662	1.02	(0.89 to 1.17)	1.02	(0.89 to 1.17)	1.02	(0.89 to 1.18)
	father only		1637	1545	1.23***	(1.11 to 1.35)	1.24***	(1.12 to 1.38)	1.28***	(1.15 to 1.42)
	both parents		1946	1800	1.25***	(1.14 to 1.38)	1.26***	(1.15 to 1.39)	1.28***	(1.16 to 1.42)
	not known^d^		224	169	—		—		—	

Hepatoblastoma	neither parent		9	1601	1.0					
	mother only		3	662	0.81	(0.22 to 2.99)	0.80	(0.21 to 2.97)	0.85	(0.23 to 3.19)
	father only		8	1545	0.92	(0.35 to 2.39)	1.10	(0.42 to 2.91)	1.23	(0.46 to 3.28)
	both parents		20	1800	1.98	(0.90 to 4.35)	2.28*	(1.02 to 5.09)	2.69*	(1.18 to 6.13)
	not known^d^		3	169	—		—		—	

* *P*<0.05,

^**^P<0.01,

*** *P*<0.001.

a Obtained from unconditional logistic regression using all controls as comparison group.

b As footnote a, adjusting for sex of child, age at death or corresponding age for controls (0–3, 4–7, 8–15y), and year of death or corresponding year for controls (1953–55, 1971–76, 1977–81).

c As footnote b, additional adjustment for social class (I, II, III, IV, V), sibship position (1, 2, 3, 4, ⩾5), age of mother at birth of survey child (<20, 20–24, 25–29, 30–34, 35–39, ⩾40y), age of father at birth of survey child (<24, 25–29, 30–34, 35–39, ≥40) and obstetric radiography (yes/no).

d One or both parents.

, together with corresponding odds ratios. Three sets of odds ratios are shown: separate analyses of parental smoking habits (A), simultaneous analyses of parental smoking habits (B), and an alternative approach related to the habits of one or both parents (C). Positive associations are evident between hepatoblastoma risks and both maternal and paternal smoking. The largest relative risk is shown in the fuller model for both parents being smokers (RR 2.69, *P*<0.05, 95% CI 1.18–6.13).

## DISCUSSION

The current analysis ignores the original individual matching but the findings for all cancers are similar to those obtained previously from analyses in which the individual matching was maintained (Sorahan *et al*, 1997b). The analyses depended on self-reported histories, and questions directed at habits either before the relevant pregnancy (1977–81 deaths) or at the time of interview (1953–55 deaths, 1971–76 deaths). There was no requirement for ex-smokers to identify themselves, and only a small percentage did so (Sorahan *et al*, 1995, 1997b). The deaths of the case children may have influenced the information supplied by case mothers. In addition, participation rates in the later phases of the OSCC were modest (Sorahan *et al*, 1995, 1997b). There is thus scope for biased comparisons of cases and controls. It is difficult to imagine, however, how a bias focused on hepatoblastoma could have been introduced. Hepatoblastoma is a rare cancer and the size of the current case series was only 43 (compared with 28 such cases in the UKCCS). This study provides considerable support, therefore, to the hypothesis that parental cigarette smoking is a risk factor for childhood hepatoblastoma (Pang *et al*, 2003; Pang and Birch, 2003). It is possible to speculate that the importance of both parents smoking in the aetiology of hepatoblastoma might arise from the combination of oxidative damage to sperm DNA (Fraga *et al*, 1996) and damage to the fetal liver from carcinogenic metabolites in the blood of the pregnant mother.
